# Retrospective analysis of traumatic triradiate cartilage injury in children

**DOI:** 10.1186/s12891-021-04565-2

**Published:** 2021-08-10

**Authors:** Yao Dong, Jinkui Wang, Jiaqiang Qin, Guoxin Nan, Yuxi Su, Bo He, Wenquan Cai, Kai Chen, Kai Gu, Xun Liang, Ge Yan, Zhongliang Wang

**Affiliations:** 1 Department of Orthopedics; Ministry of Education Key Laboratory of Child Development and Disorders, National Clinical Research Center for Child Health and Disorders (Chongqing), China International Science and Technology Cooperation base of Child development and Critical Disorders, Chongqing Key Laboratory of Pediatrics, Children’s Hospital of Chongqing Medical University, 2 ZhongShan Rd, 400013 Chongqing, P.R. China; 2 Department of Orthopedics, The First People’s Hospital of Ziyang, Sichuan, P.R. China

**Keywords:** Pelvic fracture, Triradiate cartilage, Injury, Diagnosis, Treatment, Acetabular dysplasia

## Abstract

**Background:**

To summarize and analyze the epidemiological characteristics, treatment and corresponding curative effect of triradiate cartilage injury(TCI) in children after trauma, to provide a theoretical basis for early diagnosis and improvement of treatment.

**Methods:**

The TCI was classified according to Bucholz classification, and the final curative effect was evaluated with Harris Hip Score and imaging examination during follow-up. Finally, a comprehensive analysis was made by reviewing the cases in the literature combined with the patients in our hospital.

**Results:**

A total of 15 cases (18 hips) of triradiate cartilage injuries were collected in our hospital. There was 1 hip with type I injury, nine hips with type II injury, two hips with type IV injury, one hip with type V injury and five hips with type VI injury. Among the 12 cases with complete follow-up, the bone bridge was found in or around the triradiate cartilage in 8 cases, early fusion of triradiate cartilage occurred in 5 patients, 3 cases had hip dysplasia, 4 cases had a subluxation of the femoral head, and HHS was excellent in 8 cases and good in 4 cases.

**Conclusion:**

The early diagnosis of TCI is still a difficult problem. Conservative treatment is often the first choice. The overall prognosis of acetabular fractures involving triradiate cartilage is poor. The formation of the bone bridge in triradiate cartilage usually indicates the possibility of premature closure, which may lead to severe complications of post-traumatic acetabular dysplasia and subluxation of the femoral head.

## Introduction

**Fig. 1:**
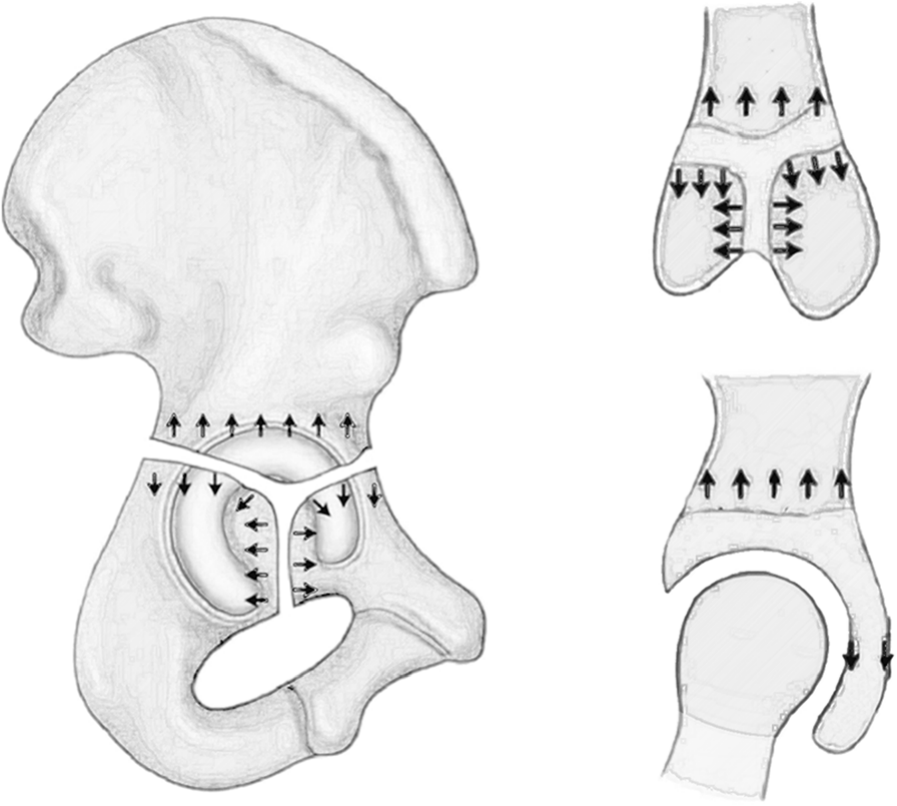
Schematic diagram of triradiate cartilage growth. The direction arrow shows the development of triradiate cartilage

Triradiate cartilage is located between the ilium, pubis and ischium. Its branches are arranged like a Y-shape, namely the iliopubic arm, iliosciatic arm, and ischiopubic arm. Different from the growth structure of long bones, each triradiate cartilage has bone growth plates on both sides, and the central zone is the secondary ossification centre, so it grows bipolar(Fig. 1). Triradiate cartilage can be divided into germinal area and hypertrophy zone. The germinal area has a large blood supply and is surrounded by a hypertrophic zone [1, 2]. The secondary ossification centre of the Triradiate cartilage usually appears around ten years old and closes at 14 years old, marking the end of acetabular development [3]. Triradiate cartilage is the main structure that determines the development of the acetabulum. The interstitial growth of the triradiate cartilage and the additional expansion of the peripheral perichondrium lead to the increase in the volume, width and height of the acetabulum. Intrachondral osteogenesis promotes the continuous growth and closure of these epiphyses [1, 2, 4, 5]. Severe Triradiate cartilage injury(TCI) will directly affect the growth and development of the acetabulum, leading to acetabular dysplasia.

TCIs often occur in high-energy pelvic injuries [6]. Acetabular fractures account for 0.8 ~ 15.2% of children’s fractures, of which about 0 ~ 11% involve cartilage [1, 6, 7]. TCI is relatively insidious, and early diagnosis is rugged and easy to miss. The current literature on TCI is mostly case reports, and its epidemiological characteristics, degree of injury, type of injury, and treatment plan are unclear. Both surgical treatment and conservative treatment are advocated. The common goal is to restore the anatomy while avoiding further damage to the triradiate cartilage blood supply, to reduce the occurrence of complications such as acetabular dysplasia and femoral head subluxation [8]. We review the literature and summarize the TCI cases in our hospital, aiming to provide experience for the early diagnosis and treatment of TCI.

## Result

From January 2010 to December 2017, a total of 191 children with pelvic fractures were admitted to our hospital. Among them, 24 children (12.57%) with acetabular fractures, 15 children (7.85%) with Triradiate cartilage injuries. These children with TCI, only 8 cases were diagnosed early, four cases were diagnosed during follow-up, and 3 cases were found during the review. Among the 15 children, 10 were males, and 5 were females, ranging from 1.4 to 9.8 years old, with an average of 5.6 years old. Among them, 4 cases were under 3 years old, 5 cases were 3 to 6 years old, and 7 cases were over 6 years old. There were 12 cases of traffic accident injuries, 2 cases of crush injuries, and 1 case of fall injuries. Among the 15 children, 3 cases involved bilateral triradiate cartilage, and 6 cases of unilateral injury, totalling 18 hips. One hip with type I injury, nine hips with type II injury, and one hip with type V injury. Seven hips cannot be classified according to the Bucholz classification, of which two hips are fracture lines that entirely through the triradiate cartilage and involve the metaphyses on both sides, similar to type IV epiphyseal injuries; 5 hips are fractures of the proximal suprapubic branch, the imaging examination did not show the triradiate cartilage involved, but in the later follow-up, it was found that the bone bridge was formed inside the triradiate cartilage, we tentatively determined it as a type VI injury. There were 7 cases with severe joint dislocation, including 3 cases with pubic symphysis diastasis(PSD), 4 cases with sacroiliac joint dislocation(SIJD), and 1 case with PSD and SIJD. Eight patients with TCI were treated conservatively, including bed rest, external brace fixation, lower extremity skin traction, and then gradually bearing weight. Seven children with severe pelvic fractures were treated with closed reduction and external stent fixation or open reduction and internal fixation(ORIF).

During the follow-up, two of the children had undergone high amputation due to severe damage to their lower limbs, and the parents were unwilling to cooperate with the follow-up, one child lost to follow-up. A total of 12 children were followed up. The follow-up time was 1.5 to 7.8 years, with an average of 5 years. The general information on admission of the children is shown in Table 1. The hip function of children was based on HHS (excellent 91–100; good 81–90; fair 71–80; poor 70 or less). Among the 12 children followed up, 8 cases were excellent, and 4 cases were good. The follow-up results are shown in Table 2. The AI, D/W, and AHI were measured by pelvic X-rays to assess the development of the acetabulum and the subluxation of the femoral head. Eight cases had bone bridges formed around triradiate cartilage, and 5 cases had triradiate cartilage premature closure, 2 cases had acetabular dysplasia, and 4 cases had femoral head subluxation.

**Table 1:** General data of 12 children with Triradiate cartilage injury

Case	Sex	Age	Side	Type	Location	Complications	Treatment
A	F	6y	L/R	II/I	Left iliopubic branch, Right pubis	PSD	Closed reduction and external stent fixation
B	M	6.2y	L	VI	Suprapubic branch	SIJD	Closed reduction and external stent fixation
C	M	1.4y	R	VI	Suprapubic branch	No	External brace fixation
D	F	3y	L/R	VI/VI	Suprapubic branch	No	Lower limb traction +External brace fixation
E	M	5.1y	R	IV	Iliosciatic branch	No	Lower limb traction + External brace fixation
F	F	2.3y	L/R	II/II	Bilateral ischiopubicbranch	PSD	Closed reduction and external stent fixation
G	M	4y	L	V	Left iliosciaticbranch	No	External brace fixation
H	M	5y	L	II	ischiopubicbranch	SIJD	Closed reduction and external stent fixation
I	M	2.2y	R	II	ischiopubicbranch	PSD	Closed reduction and external stent fixation
J	M	8.5y	R	II	ischiopubicbranch	No	Lower limb traction +External brace fixation
K	F	8.8y	L	II	Iliosciatic branch	PSD/SIJD	ORIF of L sacroiliac joint and symphysis pubis
L	F	7.8y	R	II	Iliopubic branch	No	External brace fixation
M	M	5.4y	R	II	ischiopubicbranch	No	External brace fixation
N	M	7.9y	L	VI	Suprapubic branch	No	Open reduction and external stent fixation + amputation
O	M	9.8y	L	IV	ischiopubicbranch	SIJD	External brace fixation +Open reduction and external stent fixation + amputation of pelvic fracture

*ORIF* Open reduction and internal fixation, *PSD* Pubic Symphysis Diastasis, *SIJD* Sacroiliac Joint Dislocation, *L* Left, *R* Right, *M* Male, *F* Female;

**Table 2:** fallow-up of 12 children with Triradiate cartilage injury

Case	Follow-up(mo)	Bone bridge	Premature closure	Acetabular dysplasia	Femoral head subluxation	HHS	AI(°)(L/R)	D/W(L/R)	AHI(L/R)
A	17	yes	yes	no	no	excellet	16/10	0.324/0.38	1/1
B	66	yes	no	no	no	excellet	8/22	0.359/0.319	0.93/0.96
C	77	yes	no	yes	yes	excellet	19/12	0.3/0.216	0.856/0.695
D	32	yes	no	no	no	excellet	27/25	0.266/0.288	0.795/0.81
E	56	no	no	yes	no	good	15/20	0.281/0.247	1/1
F	94	no	no	no	no	excellet	21/21	0.286/0.288	1/1
G	71	yes	yes	yes	yes	good	27/21	0.214/0.26	0.62/0.844
H	34	yes	yes	no	yes	excellet	23/13	0.336/0.294	0.64/1
I	80	no	no	no	no	excellet	24/19	0.323/0.332	1/1
J	78	yes	yes	no	yes	good	17/19	0.3/0.26*	0.77/0.67
K	80	yes	yes	no	no	good	8/18	0.344/0.327	0.871/0.755
L	36	no	no	no	no	excellet	22/14	0.306/0.313	1/1

### Typical cases

#### Case 1(patient A)

**Fig. 2:**
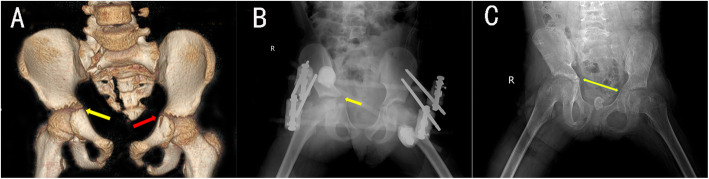
A 6-year-old girl (case A); Three-dimensional CT imaging of the pelvis revealed that the left suprapubic ramus fracture involved Y-shaped cartilage (red arrow), and the right Y-shaped cartilage gap widened (yellow arrow) (**A**); The postoperative X-ray showed that the pelvis was fixed with Orthofix external stent, and the right Y-shaped cartilage gap was still significantly widened (yellow arrow) (**B**). Seventeen months after the operation, the X-ray showed that the Y-shaped cartilage was partially closed (**C**)

A 6-year-old girl suffered a pelvic fracture after being hit by a car. CT examination showed multiple fractures of the pelvis, bilateral acetabular fractures, separation of the pubic symphysis, and sacral fractures. Triradiate cartilage on the right is a type I injury, and the left is type II injury(Fig. 2A). Closed reduction and external stent fixation were performed after the vital signs were stable(Fig. 2B). Two months after the operation, the pelvis gradually bears weight. Follow-up 17 months after the operation, the child had mild claudication, no hip joint pain, and the HHS was excellent. X-rays of the pelvis showed that the pelvis was slightly tilted and the triradiate cartilage was partially closed, but the acetabulum and femoral head contained a good relationship(Fig. 2C).

#### Case 2(patient C)

**Fig. 3:**
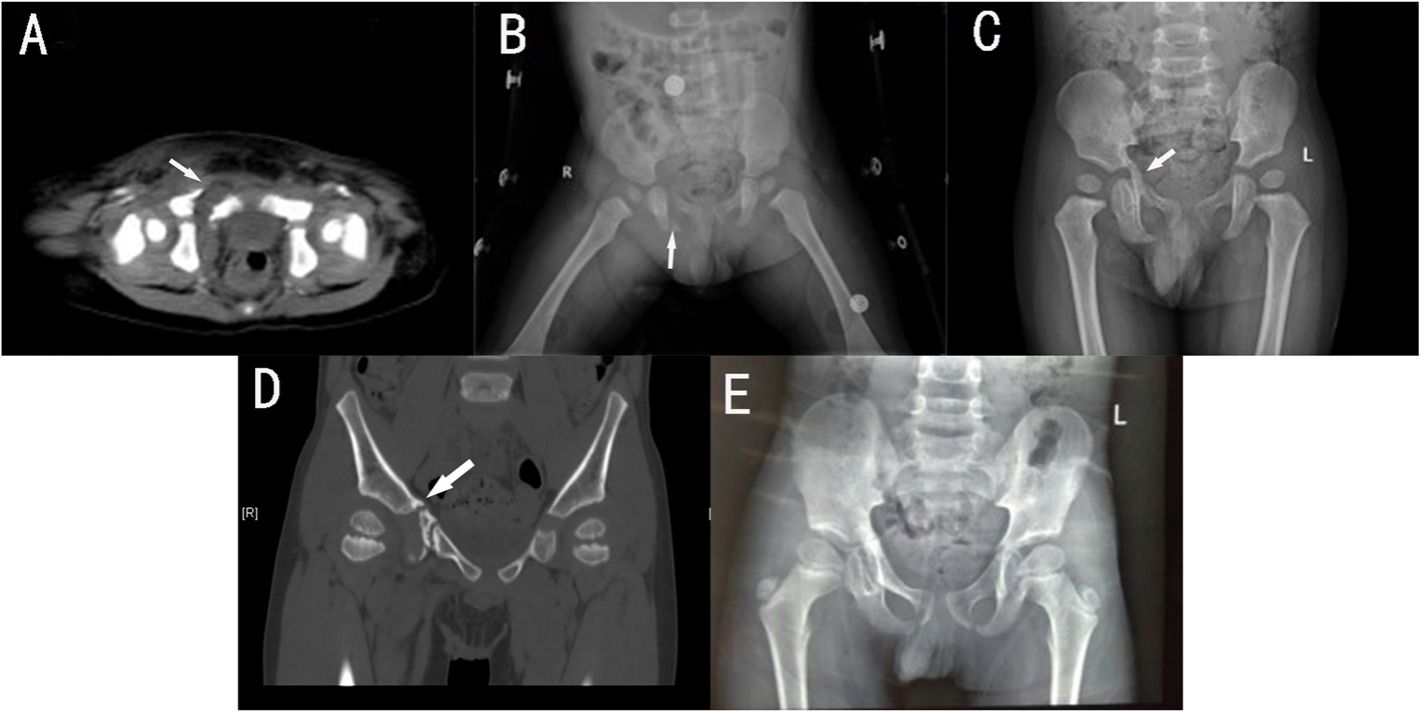
A 1.4-year-old boy (case C). X-ray and CT suggests fractures of superior and inferior ramus of pubis and right ischium (white arrow) (**A**, **B**). Two weeks later, X-ray showed a faint bony bridge formation across the medial margin of the triradiate cartilage on the right acetabulum (**C**) (white arrow). Five months after the injury, X-ray showed rupture of the original bone bridge (white arrow) (**D**). Seven years after injury, X-ray showed that the triradiate cartilage remained open, but the medial wall of the right acetabulum had thickened, the acetabulum had become shallow, and the femoral head had shifted slightly laterally (**E**)

A 1.4-year-old boy was hit by a car, resulting in a fractured pelvis, a fractured femur, and an abdominal injury. X-ray and CT examination of the pelvis revealed fractures of the upper and lower branches of the right pubic bone and the right ischia(Fig. 3A-B). Treatment includes bed rest, brace fixation of the lower limbs and pelvis. Two weeks later, the X-ray showed the formation of a hematoma at the fracture of the right suprapubic ramus and the formation of a bone bridge across the medial edge of the triradiate cartilage, and was classified as type VI TCI(Fig. 3C). Five months later, the original bone bridge was found to be broken(Fig. 3D). We believe that the fracture of the bone bridge will not further affect the development of the acetabulum, so we choose to continue conservative treatment. Seven years later, the child had mild pain in the right hip, no claudication, and an excellent HHS score. An X-ray showed that the bone bridge was broken, the inner wall of the right acetabulum became thick, the acetabulum became shallow, and the femoral head shifted to the outside(AI 12°, D/W 0.216, AHI 0.683)(Fig. 3E).

#### Case 3(patient E)

**Fig. 4:**
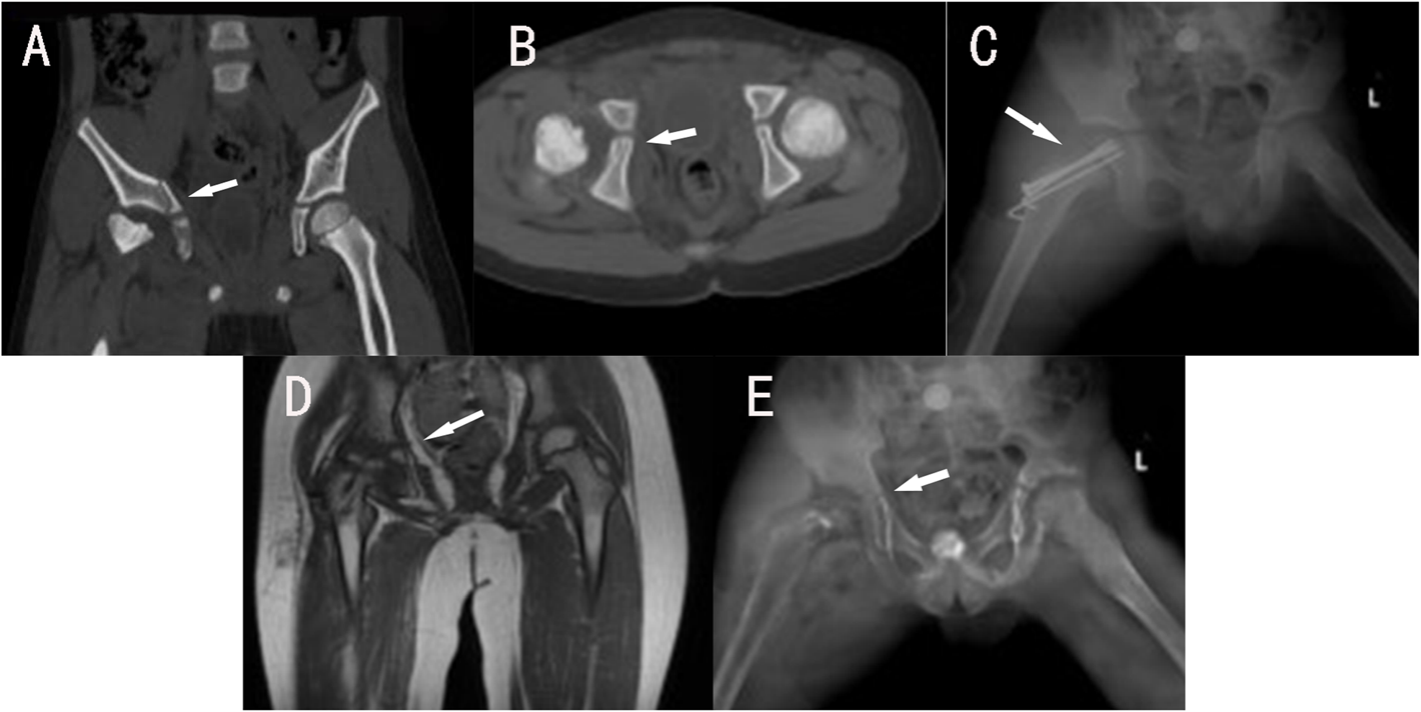
A 5.1-year-old boy(case E) was crushed by a car and caused a pelvic fracture involving right ilium and right acetabulum fractures, and a fracture of the left femur neck. CT showed a fracture line from the iliac metaphyseal through the triradiate cartilage to the ischiadic metaphyseal and was classified as type IV TCI (**A**, **B**) (white arrow). After the essential condition is stable, the right femur neck was performed ORIF (**C**). One year later, MRI showed partial necrosis and collapse of the femoral head, with the triradiate cartilage had no bone bridge formation (**D**). Four years after the operation, the patient had mild claudication with dysfunction such as squatting and stocking. X-ray showed slightly oblique acetabulum and necrosis of femoral head (**E**)

A 5.1-year-old boy was crushed by a car and caused a pelvic fracture involving right ilium and right acetabulum fractures, and a fracture of the left femur neck. CT showed a fracture line from the iliac metaphyseal through the triradiate cartilage to the ischiadic metaphyseal and was classified as type IV TCI(Fig. 4A,B). After the essential condition is stable, the right femur neck was performed ORIF(Fig. 4C). One year later, MRI showed partial necrosis and collapse of the femoral head, with the triradiate cartilage had no bone bridge formation(Fig. 4D). Four years after the operation, the patient had mild claudication with dysfunction such as squatting and stocking. X-ray showed slightly oblique acetabulum and necrosis of femoral head.

## Methods

We collected children with TCI admitted to the Children’s Hospital of Chongqing Medical University from January 2010 to December 2017. This research was approved by the Ethics Review Board of the Children’s Hospital of Chongqing Medical University. All patients or their relatives provided written informed consent to be included in this study. Inclusion criteria: 1)triradiate cartilage was not closed at the time of injury; 2)TCI after trauma (widening, narrowing, rupture, premature closure and bone bridge formation after injury); 3)Follow-up time ≥ 12 months. We collect their general information (age, gender, injury location), physical examination, imaging data, and treatment methods of these children. The children were followed up regularly, including hip joint pain, claudication, daily life and sports, hip joint range of motion, lower limb length, and pelvic tilt. Some children with X-ray, Computerized Tomography(CT) or Magnetic Resonance Imaging(MRI) examination to understand the development of acetabulum and TCI.

Bucholz et al. classified TCI according to the Salter-Harris epiphyseal injury classification [1], and we also made a preliminary classification of TCI. We evaluated the hip joint function of the child at the latest follow-up based on the Harris hip score(HHS). We assessed the development of the acetabulum by measuring the Acetabular Index (AI) and the Depth-to-Width ratio (D/W) of the acetabulum. We evaluated the subluxation of the hip by using the Head of Acetabular Index (AHI) (AI greater than 30°or D/W less than 0.25 is considered acetabular dysplasia; AHI less than 0.75 is deemed to be hip subluxation).

## Discussion

TCI in children is rare, mostly in case reports. We use “pelvic fracture” and “child” and “triradiate cartilage” or “Y cartilage” or “growth plate”or “acetabular fracture” as the keyword to review all English articles on traumatic triradiate cartilage injuries in children. We found 12 articles in total, including 29 cases, 17 males and 12 females, with an average age of 7 years old. Among them, 20 cases have a reliable classification, including 4 cases of type I, 4 cases of type II, 6 cases of type V, and 6 cases of type VI(which are classified as type V initially). Most of them received conservative treatment, and only four children underwent bone bridge resection, of which 2 had no hip pain and no acetabular dysplasia. Twenty-nine children were followed up for an average of 7 years. In 12 cases, bone bridges across triradiate cartilage were formed in the early post-injury period, appears as early as 6 weeks after injury. Nine cases had triradiate cartilage epiphysis premature closure. Acetabular dysplasia occurred in 12 cases, and 11 cases showed hip subluxation. There were also complications such as hip pain, lower limb shortening, claudication, pelvic tilt, and pelvic dysplasia (Table 3). Among children with TCI in our hospital, type II injury is the most common, but type V is the most common type reported in previous literature. It may be due to the advancement of modern medical imaging technology that fracture fragments are easier to find.

**Table 3:** Literature review of traumatic triradiate cartilage injury

References	year	cases	Age(average)	sex	side	location	type	treatment	Follow-up(mo)(average)	Bone bridge	Premature closure	Acetabular dysplasia	Femoral head subluxation
Spina1 M et al. [9]	2019	1	14y	M	R	iliosciatic arm、 ischiopubic arm	I	ORIF	2	1	No	No	No
Badina A et al. [10]	2013	3	4y	2 M/1 F	3 R	N	VI;II;VI	Bone bridge resection	6	3	1	1	1
Muhittin S et al. [11]	2008	1	8y	F	L	ischiopubic arm	II	Conservative treatment	1.7	1	1	No	No
McDonnell M et al. [12]	2007	1	13y	M	R	iliosciatic arm、 ischiopubic arm	I	Conservative treatment	2	No	No	No	No
Peterson H A et al. [13]	1997	1	5y	M	R	N	VI	Bone bridge resection	12	1	1	1	1
Pina-Medinaet,A et al. [14]	1996	1	14y	M	L	ischiopubic arm	II	ORIF	2	No	No	No	No
Trousdale R T et al. [7]	1994	5	3y	2 M/3F	2 L/3 R	N	N	Conservative treatment first, 3 Periacetabular osteotomy, 1 Chiari osteotomy	15	N	N	5	5
Heeg M et al. [4]	1988	4	4y	2 M/2 F	3 R/1 L	N	VI; VI; V; I	Conservative treatment	9	4	4	2	2
Bucholz R W et al. [1]	1982	9	10y	5 M/4 F	5 R/4 L	N	4 V;4I;1II	1 ORIF, 8 Conservative treatment	3	N	N	1	N
Alan Weisel1 et al. [15]	1980	1	4y	M	R	iliosciatic arm、 ischiopubic arm	I	Conservative treatment	0	N	N	N	N
Blair W et al. [16]	1979	1	4y	M	L	N	VI	Conservative treatment first, Chiari osteotomy after 12 years	12	1	1	1	1
Rodrigues KF et al. [17]	1973	1	3y	M	L	N	V	Conservative treatment	15	1	1	1	1

*ORIF* Open reduction and internal fixation, *N* Not available, *L* Left, *R* Right, *M* Male, *F* Female

### Classification of TCI

**Fig. 5:**
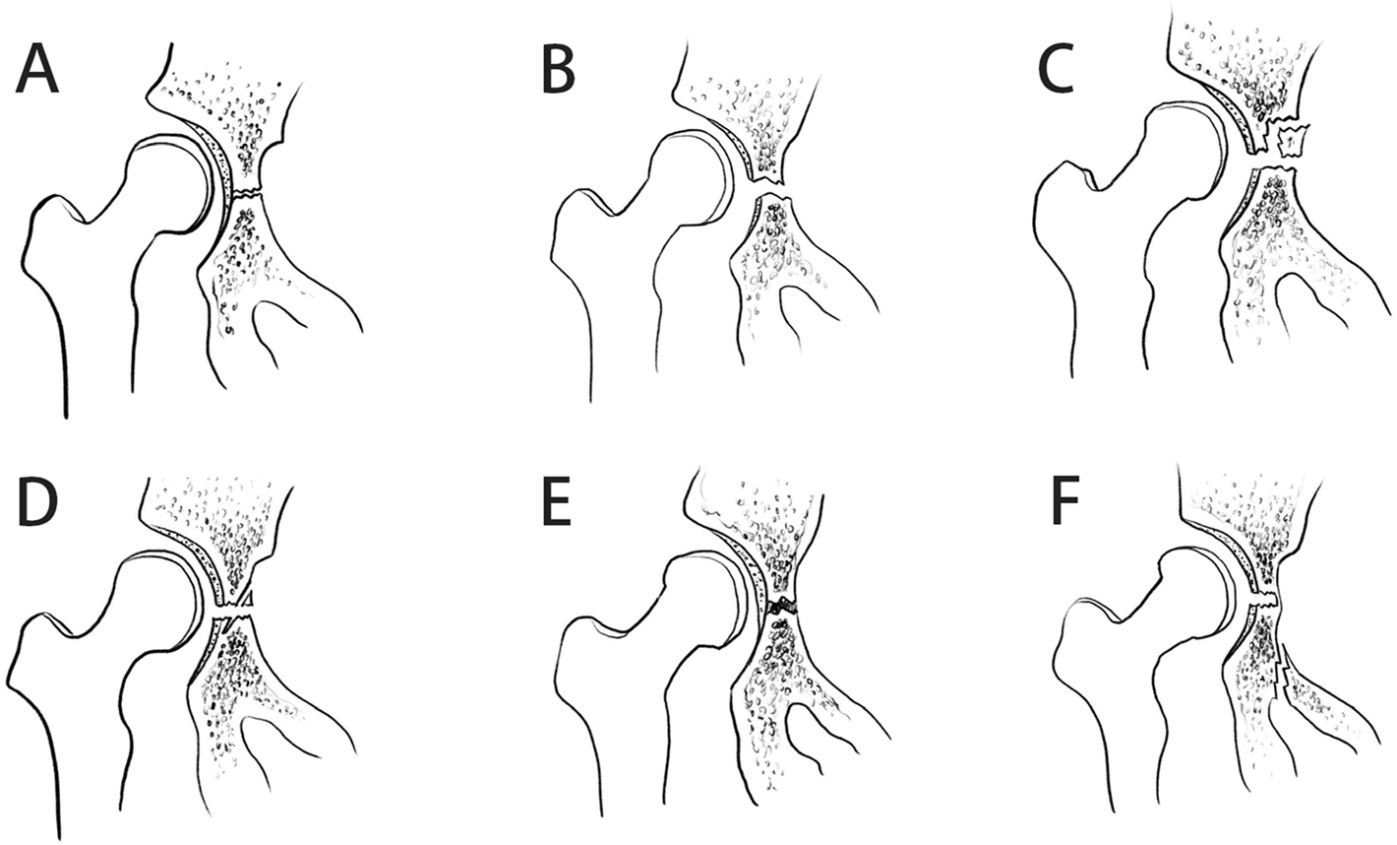
Modified Bucholz classification. **A** Normal, **B** Type I, **C** Type II, **D** Type IV, **E** Type V, **F** type VI

The Bucholz classification of TCI is based on the Salter-Harris epiphyseal classification, which is divided into I, II, and V types [1]. However, in the case of our hospital, it was found that two injuries could not be classified according to the Bucholz classification, so we propose a modified Bucholz classification(Fig. 5). The two new injury types are defined as type IV and type VI respectively; type IV is a fracture that penetrates the triradiate cartilage and the metaphysis on both sides, which is caused by vertical shear force. Type VI is a bone bridge formed by a hematoma caused by a fracture of the suprapubic branch, which was reported in several cases in the previous literature (6/29). However, they are all classified as type V. We believe that the formation of type VI bone bridge is different from type V injury in that it is formed outside instead of inside the triradiate cartilage [4].

### Diagnosis of TCI

TCI mostly occur in high-energy traumas such as car accident injuries and fall injuries, and most of them are children under ten years old. Du to triradiate cartilage gradually begins to ossify and fuse after ten years of age. More than 50% of triradiate cartilage injuries are missed in the initial diagnosis. The first missed diagnosis rate of the cases in this study is as high as 46.7% [4, 11]. Therefore, the imaging performance of TCI is essential. TCI can be manifested as widening or narrowing of the triradiate cartilage gap, small bone fragments at the iliac, pubic, and ischial metaphysis, and changes in the relationship between the ilium, ischium, and pubis. After the injury, bone bridges formation, triradiate cartilage ossification, and premature closure will be seen in TCI. TCI can be detected by X-ray if the fracture has significant displacement, such as type II or type IV. If fracture displacement is not obvious, most TCI can be detected by 3D reconstruction CT. Type I and V TCI without significant displacement may make it difficult to confirm the diagnosis. MRI of the hip joint is recommended to confirm the presence of TCI. Besides, MRI can clearly distinguish the size of the hematoma formed around the triradiate cartilage in the early stage, to predict the size of the bone bridge formation, and provide evidence for whether to perform bone bridge resection. In addition, Children with suspected TCI, who cannot be identified by radiographic examination but is accompanied by pelvic or acetabular injury, should be followed up for at least 1 year. Those who are diagnosed with TCI should be followed up until the bones mature.

### Treatment of TCI

Due to the unique development of children’s acetabulum, TCI requires individualized treatment. Both surgical treatment and conservative treatment have been reported. For unstable pelvic fractures and acetabular fractures, regardless of whether it is combined with TCI, active ORIF is recommended, which is very helpful to restore the anatomical structure of the pelvis and reduce complications [18, 19]. Most patients in the previous literature have received conservative treatment, includes bed rest and skeletal traction for 4 to 8 weeks [7]. Some authors believe that the bone bridge at the triradiate cartilage should not be treated by surgery because the location is difficult to access, and the operation may cause damage to surrounding structures [11]. Another objection to surgical treatment is that the bone bridge formed after trauma may be too large to be removed, and even after the removal, the bone bridge may still be constructed again [13]. In our case series, the degree of displacement of the acetabular fracture in children was light, and the integrity of the acetabular joint surface was maintained well. All children were treated conservatively. Two children with a small bone bridge ruptured spontaneously after 2 to 4 months. No evident acetabular dysplasia was found in the follow-up. One case with a large bone bridge broke spontaneously 5 months after the injury. However, in the later follow-up, it was found that the inner wall of the acetabulum gradually thickened, the acetabulum became shallow, and the femoral head subluxation.

We believe that the displacement greater than 2 mm or type VI triradiate cartilage injury should be treated with ORIF; other types of injuries can be treated conservatively. All children should be followed up until the triradiate cartilage is closed. Those with acetabular dysplasia should undergo pelvic osteotomy as soon as possible to improve the shape of the acetabulum and avoid the occurrence of osteoarthritis. When a bone bridge appears during follow-up, it should be determined whether to perform bone bridge resection based on factors such as the age of the child, the size of the bone bridge, and the degree of acetabular development. The age of the child is the most important influencing factor for acetabular dysplasia because the younger the generation indicates the more potential for growth of triradiate cartilage, which can cause acetabular dysplasia [12].

## Conclusion

Early diagnosis of TCI in children is quite difficult. For suspected cases, the multi-angle hip joint X-ray and CT three-dimensional reconstruction should be perfected. If necessary, MRI should be performed. For patients with clear indications for acetabular fracture surgery or type VI TCI, ORIF should be actively performed. Conservative treatment is often recommended for most triradiate cartilage injuries. The prognosis of TCI is often poor. Age, injury location, and degree of injury are important factors affecting the prognosis. Children with TCI should be followed up until the bones mature.

## Data Availability

All data generated or analysed during this study are included in this published article.

## Abbreviations

TCI: Triradiate cartilage injury
AI: Acetabular Index
AHI: Acetabular Head Index
D/W: Depth-to-width Ratio
HHS: Harris Hip Score
PSD: Pubic Symphysis Diastasis
SIJD: Sacroiliac Joint Dislocation
